# Hereditary etiology of non-syndromic sensorineural hearing loss in the Republic of North Ossetia–Alania

**DOI:** 10.7717/peerj.14514

**Published:** 2023-01-30

**Authors:** Nika Petrova, Inna Tebieva, Vitaly Kadyshev, Zalina Getoeva, Natalia Balinova, Andrey Marakhonov, Tatyana Vasilyeva, Evgeny Ginter, Sergey Kutsev, Rena Zinchenko

**Affiliations:** 1 Research Centre for Medical Genetics, Moscow, Russian Federation; 2 North Ossetian State Medical Academy of the Ministry of Health of the Russian Federation, Beslan, Russian Federation; 3 Medical and Genetic Consultation of the Republican Children’s Clinical Hospital of the Republic of North Ossetia–Alania, Vladikavkaz, Russian Federation; 4 Pravoberezhnaya Central Clinical Hospital of the Ministry of Health of the Republic of North Ossetia–Alania, Vladikavkaz, Russian Federation; 5 N. A. Semashko National Research Institute of Public Health, Moscow, Russian Federation

**Keywords:** North Ossetia–Alania, GJB2, POU3F4, MYH14, MYO15A, Non-syndromic sensorineural hearing loss

## Abstract

More than 50% of congenital hearing loss is hereditary, in which the majority form is non-syndromic. In this study we estimate the most prevalent pathogenic genetic changes in an Ossetian cohort of patients. This is useful for local public health officials to promote genetic counseling of affected families with regard to high allele frequencies of prevalent pathogenic variants and assortative mating in the community of people with hearing loss. In this study, genetic heterogeneity of hereditary non-syndromic sensorineural hearing loss (NSNHL) in a cohort of 109 patients and an assessment of the frequency of two *GJB2* gene pathogenic variants in a cohort of 349 healthy individuals from the populations of the Republic of North Ossetia–Alania (RNO–Alania) were assessed. The molecular genetic cause of NSNHL in the *GJB2* gene in RNO–Alania was confirmed in ~30% of the cases, including ~27% in Ossetians. In Russian patients, the most frequent variant is *GJB2*:c.35delG (~83%). The *GJB2*:c.358_360delGAG variant was found to be the most frequent among Ossetians (~54%). Two genetic variants in *GJB2*, c.35delG and c.358_360delGAG, accounted for 91% of *GJB2* pathogenic alleles in the Ossetian patients. A search for large genome rearrangements revealed etiological cause in two Ossetian patients, a deletion at the *POU3F4* gene locus associated with X-linked hearing loss (type DFNX2). In another Ossetian patient, a biallelic pathogenic variant in the *MYO15A* gene caused hearing loss type DFNB3 was identified, and in one Russian family a heterozygous *MYH14* gene variant associated with dominant NSNHL was found. Thus, the informative value of the diagnosis was ~37% among all patients with NSNHL from RNO–Alania and ~32% among the Ossetians. These estimates correspond to the literature data on the fraction of recessive genetic forms of hearing loss within the affected population. The importance of this study consists not only in the estimation of the most prevalent pathogenic genetic changes in the Ossetian cohort of patients which could be useful for the public health but also in the genetic counselling of the affected families with regard to the high allele frequencies of revealed pathogenic variants as well as to the assortative mating in community of people with hearing loss.

## Introduction

Congenital hearing impairment is one of the most common causes of human disability. It occurs in 1–2 out of 1,000 newborns, and more than half of these are accounted for by hereditary forms (Morton & Nance, 2006; Mehl & Thomson, 2002). According to the World Health Organization, around 360 million people (~5% of the World’s population) have hearing loss leading to disability, and 32 million of them are children (Talbi et al., 2018; The World Health Organization, 2022). In the structure of prelingual isolated hearing loss in children, sensorineural hearing loss prevails (detected in ~91% of the patients), mixed hearing loss is detected in ~7% of the patients, and conductive hearing loss is present in only in ~1.5% of cases (Petit, Levilliers & Hardelin, 2001). In the Russian Federation, 8–9 million people have various hearing impairments (All-Russian Society of the Deaf, 2022; Hereditary Hearing Loss Homepage, 2022) that manifest in different time periods of life.

Most cases (~70%) of hereditary hearing loss (HHL) are non-syndromic forms characterized by wide locus and allelic heterogeneity. More than 6,000 pathogenic variants in more than 120 genes have been identified to date. In the structure of HHL forms, those of autosomal recessive (AR) type of inheritance prevail (~70–80%), autosomal dominant forms (AD) account for up to 20%, X-linked and mitochondrial forms being relatively rare at ~1–2% and ~1% respectively (Talbi et al., 2018; Petit, Levilliers & Hardelin, 2001; Online Mendelian Inheritance in Man, 2022). The same genes can be involved in either AD or AR hearing loss and contribute to disability with digenic mode of inheritance (Morton & Nance, 2006; Talbi et al., 2018; Petit, Levilliers & Hardelin, 2001). About 20–30% of hereditary hearing disorders are detected as part of various hereditary syndromes, *i.e*., hearing loss is accompanied by damage to other organs and systems, the other damage not always being simultaneously manifested (Morton & Nance, 2006; Talbi et al., 2018; Petit, Levilliers & Hardelin, 2001). Early detection of hearing impairment and subsequent clinical and precise DNA diagnostics of the genetic forms of hearing loss allows the cause of the disease to be determined at early age in the patients. Timeliness in taking measures to rehabilitate children with hearing impairment should contribute to the success of their social adaptation.

Non-syndromic sensorineural hearing loss (NSNHL) occurs due to damage to the organs of the inner ear, the auditory nerve, or the center in the brain that is responsible for the perception of sound. Mapping the genes responsible for the occurrence of NSNHL was a breakthrough in understanding the mechanisms of the occurrence and inheritance of hearing loss. Mutations in the *GJB2* gene have been shown to be the most common cause of NSNHL (OMIM #220290) (Kenneson, Van Naarden Braun & Boyle, 2002). On average, about half of the cases of AR NSNHL in European populations are associated with mutations in the *GJB2* gene (Kenneson, Van Naarden Braun & Boyle, 2002). The highest contribution of pathogenic variants of the *GJB2* gene is shown in European countries (~27% of the cases), whereas in African countries it is much smaller (~6%) (Chan & Chang, 2014). In most European countries the most common *GJB2* pathogenic variant in patients with AR NSNHL is c.35delG. (Chan & Chang, 2014). Variant c.35delG prevails among Russians with NSNHL, accounting for up to 80% of mutant alleles of the *GJB2* gene (Bliznetz et al., 2012). Pathogenic variants in the *STRC*, *USH2A*, *SLC26A4*, *MYO7A*, *OTOF*, *MYO15A*, and *TECTA* genes are less frequent but occur in Europe and elsewhere in the world. Mutations in other genes have been found in rare cases (Del Castillo et al., 2022).

Our previous studies have shown that both allelic and locus heterogeneity genetic causes of NSNHL are observed in populations and ethnic groups of the Russian Federation: in Mari-El Republic (Zinchenko et al., 2007a); Udmurt Republic (Zinchenko et al., 2007b); Chuvash Republic (Zinchenko et al., 2007c); Karachay–Cherkess Republic (Petrina et al., 2017); Rostov region (Petrina et al., 2018; Shokarev et al., 2005); Kirov region (Sharonova, Osetrova & Zinchenko, 2009; Zinchenko, Osetrova & Sharonova, 2012); and in the Nogai population (Zinchenko et al., 2018; Petrina et al., 2020). The following mutation rates of c.35delG were found: Chuvash, 0.78%; Bashkirs, 0.25%; Karachay, 0.14%; Udmurts, 0.25%; Russians in general, ~0.04–0.05% (1:2,000–2,500 newborns). Sequencing of the *GJB2* gene has made it possible to identify additionally only one or two mutations that have very low population frequencies. Further, in our works locus heterogeneity was determined for Chuvash, for whom NSNHL associated with the *GIPC3* gene variants was found. The frequency of *GIPC3* genetic variant c.245G>A in Chuvash NSNHL patients was ~21%, and the frequency of carrying variant c.245G>A was 1/44 in the Chuvash, the population frequency being 0.01143, *i.e*., more than 1% (Petrova et al., 2021).

The main diagnostic protocols and algorithms developed in the Russian Federation are based on general population data concerning the title ethnic group of Russia (“Russians”), who make up more than 80% of the population of the country. Given that the population of the Russian Federation is represented by many nationalities, it is necessary to study the ethnic characteristics of molecular diagnostics with subsequent optimization of existing research protocols, and this is currently being carried out by workers of the Laboratory of Genetic Epidemiology of the Research Centre for Medical Genetics (Zinchenko et al., 2007a; Petrova et al., 2021).

In this work the results of the study of the genetic and allelic genetic heterogeneity of hereditary non-syndromic hearing loss (NSNHL) in the Republic of North Ossetia–Alania (RNO–Alania) is reported and the frequencies of a number of *GJB2* gene variants and their heterozygous carrier rate in healthy Ossetians are assessed.

## Materials and Methods

The material studied was DNA isolated from blood samples of NSNHL patients and healthy Ossetians from RNO–Alania. Informed consent was obtained from all participants or their legal guardians. The study was approved by the Ethics Committee of the Research Centre for Medical Genetics. The study was conducted in accordance with the Declaration of Helsinki and was approved by the Institutional Ethics Committee of the Research Centre for Medical Genetics (protocol No. 5 dated December 20, 2010).

### Characteristic of NSNHL patients

The cohort of patients with hearing loss from RNO–Alania consisted of 109 individuals from 93 unrelated families: 83 Ossetian: 12 Russian: three Ingush: two Avar; nine people of other ethnicities. The ethnicity of the participants was determined upon completion of a questionnaire that gathered information about three generations of direct relatives from both parents.

All of the studied patients had prelingual bilateral sensorineural hearing loss of degrees 2–4 or complete deafness as determined by an audiologist. There were no known external environmental risk factors for the development of hearing loss in the anamneses of the patients. The age of the patients ranged from 0.5 to 68 years (mean age 19.87 ± 17.10 years); the male/female sex ratio was 58/53. The diagnosis was made on the basis of the clinical picture in the hearing center of Vladikavkaz, RNO–Alania. The diagnosis was established upon birth (in ~73% cases) or within the 1^st^ year of life. Patients were also examined by a geneticist at the Research Centre for Medical Genetics (Moscow) to exclude syndromic forms of sensorineural hearing loss.

### Characteristics of the cohort of healthy individuals

The cohort of healthy individuals consisted of 349 North Ossetians living in the city of Vladikavkaz as well as in the Prigorodny, Alagirsky, Ardonsky, Digorsky, and Irafsky Districts. Ethnic origin to the third generation was determined when compiling the questionnaire of informed consent to participate in the study.

### DNA isolation

DNA from peripheral blood was isolated using the Wizard Genomic DNA Purification Kit (Promega, Madison, WI, USA) in accordance with the manufacturer’s recommendations.

## Molecular genetic testing

### Search for point mutations in the *GJB2*, *GJB6*, and *GJB3* genes

The presence of the c.35delG variant in the *GJB2* gene was determined by amplified fragments length analysis (AFLP). Fragments containing the first and second exons of the *GJB2* gene as well as coding exons of the *GJB6* and *GJB3* genes were subjected to Sanger sequencing.

In the cohort of healthy individuals, the c.35delG and c.358_360delGAG variants in the *GJB2* gene were screened by AFLP using the primer sequences shown in Table 1.

**Table 1:** Primers for *GJB2*:c.35delG testing and sequencing of the *GJB2*, *GJB3* and *GJB6* genes.

Gene/variant or exon	Forward primer sequence (5′→3′)	Reverse primer sequence (5′→3′)
GJB2/c.35delG	CTTTTCCAGAGCAAACCGCCC	TGCTGGTGGAGTGTTTGTTCAC
GJB2/c.358_360delGAG	GCAGCTGATCTTCGTGTCCA	GCTTCGAAGATGACCCGGAA
GJB2/exon 1	CGTAACTTTCCCAGTCTCCGA	GCCCAAGGACGTGTGTTG
GJB2/exon 2	GTTCTGTCCTAGCTAGTGATT	GGTTGCCTCATCCCTCTCAT
GJB3/exon 2 part 1	CGTTGTGAGTATTGAACAAGTCAGAACTCAG	GTTGATCCCTTCCTGGTTA
GJB3/exon 2 part 2	CTCTGCTACCTCATCTGCCA	GTTGATCCCTTCCTGGTTGA
GJB6/exon 2 part 1	CTTTCAGGGTGGGCATTCCT	AGCACAACTCTGCCACGTTA
GJB6/exon 2 part 2	CTTCGTCTGCAACACACTGC	GCAATGCTCCTTTGTCAAGCA

### Search for large genome rearrangements associated with NSNHL

Copy number variations (CNVs) in causative genome loci were analyzed by multiplex ligase-dependent probe amplification (MLPA) analysis using the SALSA MLPA P163-GJB-WFS1-POU3F4 kit (MRC-Holland, Amsterdam, The Netherlands). The MLPA results were analyzed using the Coffalyser.Net program software (MRC-Holland). The set of probes for the MLPA analysis P163-GJB-WFS1-POU3F4 contains probes for each of the genes: *GJB3* (1p34.3); *WFS1* (4p16.1); *GJB2* (13q12.11); *GJB6* (13q12.11); *POU3F4* (Xq21.1). The CNVs are named according to the ISNC-2020 recommendations using the GRCh37/hg19 human genome reference and the LiftOver tool (UCSC Genome Browser; https://genome.ucsc.edu/).

### Whole-exome sequencing

The DNA samples from two patients were subjected to whole-exome sequencing (WES) with an BGISEQ-500 instrument using pair-end readings with a length of 2 × 100 bp and average on-target coverage of 75× with the MGIEasy Exome Capture V4 reagents (MGI, Shenzhen, China) for library preparation by Genomed Ltd, Moscow, Russia. Bioinformatic analysis was performed using an in-house software pipeline designed to detect both single-nucleotide variants (SNVs) and copy number variations (CNVs) as described earlier (Marakhonov et al., 2020). The results were further filtered for functional consequences and population frequencies (gnomAD AF <0.5% and <0.1% for recessive and dominant genes, respectively) as well as for clinical relevance according to the Human Phenotype Ontology database (Köhler et al., 2019). The pathogenicity status of the identified DNA sequence variants was established based on the recommendations of the American College of Medical Genetics and the Association of Molecular Pathology (Richards et al., 2015).

### Analysis of variants in the *MYO15A* gene

The SNVs in the *MYO15A* gene detected during WES were analyzed by bidirectional Sanger sequencing of two fragments of the first coding exon and a fragment containing exon 18. Specific primers were developed (Table 2) for amplification and sequencing of fragments of the *MYO15A* gene (isoform NM_016239.4).

**Table 2:** Primers for validation of *MYO15A* gene variants.

Variant	Forward primer sequence (5′→3′)	Reverse primer sequence (5′→3′)
c.823G>C	CTACTACGACCGGCAGTCAC	GTCATAGGGTGGGTATGGCG
c.3576G>A	CCTGTAGTCTTCGCTGGTCC	CCCCAACTTACAGACCCAGAG
c.5192C>T	GGAGGATCCAGTCCCTCCTA	TTTAGGGCGGAGCCAAGCTA

### Statistical analysis

The frequency of identified alleles was calculated according to the formula: *p*_*i*_ *= n*_*i*_*/n*, where *n*_*i*_ is the number of *i*-th alleles and *n* is the sample size (Zhivotovsky, 1991). The population frequencies of variants in different cohorts were compared using Fisher’s exact test according to a generally accepted methodology (Zhivotovsky, 1991).

## Results

During this genetic epidemiological study of the population of RNO–Alania, 117 patients with NSNHL from 100 unrelated families were revealed. Of these, 115 patients from 96 families of Ossetian origin were identified. Considering the total size of the Ossetian population in RNO–Alania (366,748) according to the All-Russia Census, 2010, this number of patients gives us the prevalence of NSNHL of 1/3,189 in the population (95% confidence interval [1/2,657–1/3,863]) (or 31.36 per 100,000 population; 95% confidence interval [25.89–37.64] per 100,000). These prevalence numbers are in accordance with global estimates (Morton & Nance, 2006; Mehl & Thomson, 2002). Of these 117 patients, 109 individuals from 93 unrelated families (83 Ossetians, 12 Russians, three Ingush, two Avar, and nine people of other ethnicity) were available for the genetic analysis.

In the studied cohort of NSNHL patients from RNO–Alania, ~30% of the cases (33/109) were caused by pathogenic variants in the *GJB2* gene: ~27% in Ossetians (22/83); half in Russians (6/12) (Table 3). The most frequent genotype in the total cohort was the homozygous genotype *GJB2*:c.[35delG];[35delG] identified in ~39% (13/33): five Ossetian; four Russian; two Ingush; two others. The second most common genotype in the total cohort was the homozygous genotype *GJB2*:c.[358_360delGAG];[358_360delGAG] identified in ~27% (9/33), with all of the patients of that genotype being Ossetians. This genotype was the most frequent at ~40% (9/22) in the Ossetians. The second most common genotype was the compound heterozygous GJB2:c.[35delG];[358_360delGAG] found in ~27% (6/22). Five patients had only one pathogenic *GJB2* allele. No pathogenic variants in the coding regions of the *GJB3* and *GJB6* genes were detected in NSNHL patients from that cohort.

**Table 3:** Results of molecular testing in NSNHL patients from RNO–Alania.

Genotype	Ossetians	Russians	Ingush	Avars	Others	Total sample
*GJB2*:c.[35delG];[35delG]	5	4	2	–	2*	**13**
*GJB2*:c.[358_360delGAG];[358_360delGAG]	9	–	–	–	–	**9**
*GJB2*:с.[35delG];[358_360delGAG]	6	1	–	–	–	**7**
*GJB2*:c.[35delG];[−23+1G>A]	1	–	–	1	–	**2**
*GJB2*:c.[35delG];[290dupA]	1	–	–	–	–	**1**
*GJB2*:c.[35delG];[95G>A]	–	1	–	–	–	**1**
*GJB2*:c.[35delG];[=]	–	–	–	1	–	**1**
*GJB2*:с.[358_360delGAG];[=]	1	–	–	–	–	**1**
rsa[GRCh37] Xq21.1(81789851×1,81841838_81842142×0,81979450×1); *GJB2*:с.[358_360delGAG];[=]	1	–	–	–	–	**1**
*MYO15A*:c.[3576G>A];[5192T>C]; *GJB2*:c.[313_326delAAGTTCATCAAGGG];[=]	1	–	–	–	–	**1**
*GJB2*:c.[313_326delAAGTTCATCAAGGG];[=]	1	–	–	–	–	**1**
rsa[GRCh37] Xq21.1(81789851×1,81841838_81842142×0,81979450×1)	1	–	–	–	–	**1**
*MYH14*:c.[500G>A];[=]	–	1	–	–	–	**1**
ni/ni	56	5	1	–	7**	**69**
**In total**	**83**	**12**	**3**	**2**	**9**	**109**

**Notes:**

^1^ni; Not identified.

* Patients from Russian inter-ethnic marriages (Russian × Ossetian, Chechen × Russian).

** Patients from other marriages (1 Armenian × Armenian, 1 Azerbaijani × Ossetian, 1 Belorussian × Russian, 3 Gipsy × Gipsy, 1 Korean × Korean).

The frequency of the *GJB2*:c.358_360delGAG and the *GJB2*:c.35delG variants was analyzed in 349 healthy individuals from the Ossetian population, which included 246 Iron-Ossetians, 45 Digor-Ossetians, and 17 Kudar-Ossetians (Table 4).

**Table 4:** Frequency distribution of the *GJB2*:c.358_360delGAG and *GJB2*:c.35delG variants in ethnic groups of Russian Federation.

Ethnic group	c.35delG allele count/number of tested alleles (frequency)	*p*-value	c.358_360delGAG allele count/number of tested alleles (frequency)	*p*-value
Ossetians	5/698 (0.0072)		9/698 (0.0129)	
Ossetians–Irons	3/492 (0.0061)		6/492 (0.0122)	
Ossetians–Digors	0/90		1/90 (0.0111)	
Ossetians–Kydars	0/34		0/34	
Karachay*	1/740 (0.0014)	0.1142		
Nogai*	1/244 (0.0041)	1.0000		
Circirsians*	2/230 (0.0087)	0.6855		
Abaza*	4/274 (0.0146)	0.2793		
Ingush*	3/302 (0.0099)	0.7037	0/302	0.0643
Chechens (Chechnya)*	1/284 (0.0035)	0.6787	2/284 (0.0070)	0.7386
Chechens (Ingushetia)*	0/180	0.5894	1/180 (0.0055)	0.6965
Tatars*	18/1420 (0.0127)	0.3536		
Bashkirs*	2/792 (0.0025)	0.2630		
Chuvash	6/768 (0.0078)	1.0000		
Udmurts*	3/1184 (0.0025)	0.1557		
Mary*	9/804 (0.0112)	0.5921		
Russians (Rostov)*	19/1320 (0.0144)	0.2265		
Russians (Kirov)*	8/412 (0.0194)	0.1224		
Russians (Pskov)*	2/204 (0.0102)	0.7054		

**Note:**

* Variant frequencies were calculated from references (Zinchenko et al., 2007a, 2007b, 2007c; Petrina et al., 2017; Shokarev et al., 2005; Sharonova, Osetrova & Zinchenko, 2009; Zinchenko, Osetrova & Sharonova, 2012; Zinchenko et al., 2018; Shearer et al., 2013).

Six heterozygous carriers of variant *GJB2*:c.358_360delGAG and three heterozygous carriers of variant *GJB2*:c.35delG were identified in the Iron-Ossetians, and only one carrier of variant *GJB2*:c.358_360delGAG was found among Digor-Ossetians (but differences of the frequencies of these variants were not significant) (Table 4).

Large copy number variations (CNVs) were analyzed by MLPA in patients who were negative or carried only one pathogenic *GJB2* variant. Pathogenic hemizygous deletion rsa[GRCh37] Xq21.1(81789851×1,81841838_81842142×0,81979450×1) affecting the upstream regulatory region of the *POU3F4* gene was revealed in two Ossetian patients (Table 3).

Due to the relatively low proportion of patients who had a molecular cause established at the first stage, whole-exome DNA sequencing of two NSNHL patients was performed. In the Ossetian patients, two probably pathogenic heterozygous variants in the *MYO15A* gene were identified–NM_016239.4(*MYO15A*):c.3576G>A, p.(Trp1192Ter) and c.5192T>C, p.(Phe1731Ser) (see Table 3). In a Russian patient, the NM_024729.3(*MYH14*):c.500G>A, p.(Arg167His) variant in the *MYH14* gene was revealed (Table 3).

## Discussion

Non-syndromic sensorineural hearing loss (NSNHL; OMIM PS220290) is a group of disabilities in which hearing loss occurs due to damage to the organs of the inner ear, the auditory nerve, or the center in the brain that is responsible for the perception of sound. About 75% of all cases of hereditary hearing loss are related to recessive non-syndromic hearing disorders. Considering the key role of the *GJB2*, *GJB6*, and *GJB6* connexin genes in the development of NSNHL, analysis for the c.35delG variant and sequencing of the non-coding and coding exons of the *GJB2* gene was performed in the first step of the study. Then point mutations in coding exons of the *GJB3* and *GJB6* genes were analyzed. The CNVs of the *GJB2*, *GJB3*, *GJB6*, *WFS1*, *POU3F4* loci were searched by MLPA analysis in patients negative for intragenic mutations in the *GJB2*, *GJB6* and *GJB3* genes.

### Spectrum of *GJB2* variants associated with hereditary hearing loss in RNO–Alania

In the total patient cohort, six different pathogenic variants of the *GJB2* gene were identified (Tables 3 and 5). The most frequent mutation causing NSNHL in many European populations, *GJB2*:c.35delG, accounted for a significant proportion of the identified alleles in the total patient cohort (~53%, 38/71): in Russian patients (~83%, 10/12); in Ossetian patients (~38%, 18/48). Hence, in Ossetian patients the frequency of the *GJB2* variant c.35delG was significantly lower than in the Russian patients (*p* = 0.0076) (Table 5).

**Table 5:** Variant count and proportion (%) of identified GJB2 gene variants in NSNHL patients from RNO–Alania.

Variant	Total (%)	Ossetians (%)	Russians (%)
c.35delG	38 (53.52)	18 (37.50)	10 (83.33)
c.358_360delGAG	27 (38.03)	26 (5.42)	1 (8.33)
c.313_326delAAGTTCATCAAGGG	2 (2.82)	2 (4.16)	
c.-23+1G>A	2 (2.82)	1 (2.08)	
c.290dupA	1 (1.41)	1 (2.08)	
c.95G>A, p.(Arg32His)	1 (0.01)		1 (0.08)
In total	71	48	12

The variant *GJB2*:c.358_360delGAG was the second most frequent in the total patient cohort (~38%; 27/71) and it was the most frequent variant in Ossetian patients (~54%; 26/48) of the identified alleles (Table 5).

The in-frame deletion variant *GJB2*:c.358_360delGAG, p.(Glu120del), which does not change the reading frame, leads to the loss of one amino acid. A functional study showed that GJB2:p.Glu120del cannot form homotypic slit channels, which leads to the loss of their conductivity (Bruzzone et al., 2003). This variant has been described in at least 20 patients with hearing loss including 7 homozygotes and 12 compound heterozygotes (Mani et al., 2009; The National Center for Biotechnology Information, 2022). This variant was classified as pathogenic (ClinVar Variation ID: 17006) (The National Center for Biotechnology Information, 2022).

According to Bliznetz and coauthors (Bliznetz et al., 2017), the variant *GJB2*:c.358_360delGAG was the sixth most common variant of the *GJB2* gene (~0.9%) in NSNHL patients from the Russian Federation, but the ethnicity of patients carrying this variant was not presented in that report. The *GJB2*:c.358_360delGAG variant was found in healthy Chechen individuals from the Republic of Chechnya and the Republic of Ingushetia (Bliznetz et al., 2017). In the study of NSNHL in the Karachay–Cherkess Republic, the *GJB2*:c.358_360delGAG variant was found in four patients from three families (Circassians and Kabardians) (Petrina et al., 2017). The geographical proximity of the studied regions and the high level of migration in the historical past suggest a common source of origin of this variant among the North Caucasian peoples.

Comparing the frequencies of the *GJB2*:c.35delG and *GJB2*:c.358_360delGAG variants, we found no statistically significant difference between Ossetians and the studied populations of the North Caucasian and Volga–Ural regions (Table 4).

Two variants, *GJB2*:c.358_360delGAG and *GJB2*:c.35delG, accounted for a large proportion of *GJB2*-associated NSNHL in Ossetians. Their total share was ~92% of the identified alleles (Table 5). Therefore, to evaluate the frequency of NSNHL associated with the *GJB2* gene in the Ossetian population, it was sufficient to test the variants c.358_360deGAG and c.35delG. A cohort of 349 healthy Ossetians was tested for the two common pathogenic *GJB2* variants (Table 4). Allele frequency of the *GJB2*:c.35delG variant was 0.0072, that of the *GJB2*:c.358_360delGAG was 0.0129, so their summed frequency is 0.0201. According to the Hardy–Weinberg equilibrium, the frequency of all pathogenic alleles of the *GJB2* gene (*q*) should be 0.0219 ((0.0201 × 100%)/91.67%); the frequency of hearing loss due to the variants of the *GJB2* gene (*q*^2^) in Ossetians should be 0.0004796 (1/2,085 of population).

### *GJB2*-negative hearing loss in RNO–Alania

For ~70% of NSNHL patients from the total patient cohort (78/111), in ~75% (63/84) of Ossetians and half (6/12) of Russians the molecular cause of hearing loss was not related to the *GJB2* gene (Table 3). In five patients (four of them Ossetians), only one heterozygous pathogenic variant in the *GJB2* gene was detected, two patients had the *GJB2*:c.313_326delAAGTTCATCAAGGG variant, two had the *GJB2*:c.358_360delGAG variant, and one Avar patient carried the *GJB2*:c.35delG variant. Hearing loss in those patients was apparently not associated with the *GJB2* gene, and the heterozygous carrier state was due to the relatively high population frequency of *GJB2* variants. Indeed, for two of those five patients it was possible to identify the molecular cause of hearing loss: in one, a carrier of the variant *GJB2*:c.358_360delGAG, the cause was found when analyzing CNVs; in the second, a carrier of the *GJB2*:c.313_326delAAGTTCATCAAGGG variant, the cause was found through whole exome sequencing. In addition, the latter patient’s affected sibling was found not to have the *GJB2*:c.313_326delAAGTTCATCAAGGG variant.

### Large genome rearrangements causing X-linked deafness

The analysis of large genome rearrangements revealed pathogenic CNVs associated with Х-linked deafness in two unrelated male patients. They were Ossetians and represented sporadic cases of hearing impairment.

In a male patient, a heterozygous carrier of the *GJB2*:c.358_360delGAG variant, hearing loss was found to be caused by hemizygous deletion rsa[GRCh37] Xq21.1(81789851× 1,81841838_81842142×0,81979450×1) found by MLPA analysis. The patient’s healthy mother was a heterozygous carrier of both of the variants carried by the proband, the rearrangement and the *GJB2*:c.358_360delGAG variant. A healthy sister was a heterozygous carrier of the *GJB2*:c.358_360delGAG variant.

A deletion found by the same MLPA probes was identified in another male patient in whom no pathogenic variant was found in the *GJB2*, *GJB3* and *GJB6* genes. Thus, X-linked hearing loss was found in two male Ossetian patients, and this accounted for ~6% of the cases (2/33).

The abovementioned chromosome deletion located in the Xq21.1 region removed a distal *cis*-regulatory region ~920 kb upstream from the *POU3F4* gene, and it did not affect the coding sequence. Exact chromosome breakpoints were not determined. Size could vary from 300 bp to 200,000 bp. Previously, two other deletions in the same area (~8 and ~200 kb in size) were identified in patients from Europe. They were associated with non-syndromic hearing loss (OMIM #304400; DFNX2) (The National Center for Biotechnology Information, 2022). Observations of similar chromosome region deletions in patients from different populations from West Europe and the North Caucasus indicate that the Xq21 region with its multiple conserved non-coding sequences is a hotspot for chromosome breaks. Earlier, Petrina and coauthors (Petrina et al., 2020) revealed the variant c.907C>T in the *POU3F4* gene as a cause of X-linked hearing loss in a Nogai family from the Karachay–Cherkess Republic. These findings are the reason to include Xq21 deletion screening as well as target Sanger sequencing of the *POU3F4* gene in the protocols of routine molecular genetic analysis of inherited deafness in the Russian Federation.

### Search for disability-causing variants by NGS analysis

Due to the relatively low proportion of patients for whom a molecular cause was established in the first stage, whole-exome DNA sequencing (WES) was performed for two NSNHL patients.

One of these patients was from a family with one affected sibling and healthy parents, and they are ethnic Iron-Ossetians who were born of a non-consanguineous marriage. Three single nucleotide variants had been identified in the *MYO15A* gene, each in a heterozygous state. The first two were in exon 1 – c.823G>C, p.(Gly275Arg) and c.3576G>A, p.(Trp1192Ter). The third was in exon 18 – c.5192T>C, p.(Phe1731Ser). Of the three identified variants, only the c.823G>C (rs183969516) variant was registered in the gnomAD database with frequency 0.0014384 (The Genome Aggregation Database, 2022). It had been found in one study of NSNHL patients from the United States and was regarded as variant of unknown clinical significance (Shearer et al., 2013). The two other pathogenic variants were identified for the first time. The results of the pathogenicity prediction for the new variants are included in Table S1.

The NM_016239.4(*MYO15A*):c.3576G>A, p.(Trp1192Ter) variant leads to creation of a premature stop codon and can be regarded as probably pathogenic. The NM_016239.4(*MYO15A*):c.5192T>C, p.(Phe1731Ser) variant is a missense mutation, and the gnomAD database contained two missense variants that result from substitutions in neighboring nucleotides: c.5191T>C (p.Phe1731Leu; rs1459406061; found on one of 248,934 alleles with frequency 0.000004017) and c.5193C>T (p.Phe1731Phe; rs767426819; found on 18 of 280,172 alleles with frequency 0.00006424), the pathogenicity of the latter variant being considered contradictory (The National Center for Biotechnology Information, 2022).

Mutations of myosin XVA encoded by the *MYO15A* gene are the cause of severe congenital deafness type DFNB3 (The National Center for Biotechnology Information, 2022). The full-size transcripts of myosin XVA contain 66 exons >12 thousand bp long and encode a 365-kDa protein that differs from other myosins by the presence of a very long 1,200-a.a. N-terminal region preceding the conservative motor domain. The tail regions of myosin XVA protein contain two MyTH4 domains, two regions similar to the membrane-coupled FERM domain, and a putative SH3 domain. Northern-blot analysis showed that myosin XVA is expressed in the pituitary gland in both humans and mice. In *in situ* hybridization experiments, myosin XVA transcripts were observed in areas corresponding to the sensory epithelium of the cochlea and the vestibular system in the developing inner ear of mice. Immuno-staining of the organ of Corti in adult mouses showed that myosin XVA protein is concentrated in the cuticle plate and stereocilia of the cochlear sensory hair cells. These results indicate a probable role of myosin XVA in the formation or maintenance of unique, actin-rich structures of sensory hair cells of the inner ear (Liang et al., 1999).

To confirm the diagnosis, we performed segregation analysis of all three identified *MYO15A* variants in the proband’s family (Table 6). All three variants were confirmed by Sanger sequencing in the proband and were found in his affected sibling, each variant being in the heterozygous state, and the healthy mother was found to be a heterozygous carrier of the two variants, c.823G>C and c.5192T>C, *i.e*., those variants making up a single complex allele of the *MYO15A* gene.

**Table 6:** Validation of single nucleotide variants and confirmation of the diagnosis in a family with *MYO15A*-associated deafness.

Variant in NM_016239.4 (*MYO15A*)	Proband	Healthy mother	Affected sibs
c.823G>C	G/C	G/C	G/C
c.3576G>A	G/A	G/G	G/A
c.5192T>C	T/C	T/C	T/C

Next, we searched for the three described *MYO15A* variants in 54 patients with NSNHL in whom the molecular cause of the disease had not been identified. The c.823G>C variant in heterozygous state was detected in four patients with frequency 0.03704 (4/108). Variants c.3576G>A and c.5192T>C were not found. With a high degree of probability, the c.823G>C variant could not be considered as pathogenic since its frequency in the population exceeds 1% according to the gnomAD database (The Genome Aggregation Database, 2022). Thus, in the affected family, c.3576G>A and c.5192T>C variants should be considered as causative.

In a Russian family with two affected patients, WES was performed for one of the patients. The variant NM_024729.3(*MYH14*):c.500G>A (rs776632941), p.(Arg167His) in heterozygous state was identified. Sanger sequencing of the *MYH14* gene fragment confirmed the heterozygosity for the variant in the proband and his affected father. This variant was present in the gnomAD database, and it was found on five alleles from 280,642 with frequency 0.0000178 among healthy people (The Genome Aggregation Database, 2022), but this was not mentioned in the ClinVar dataset as a pathogenic mutation (The National Center for Biotechnology Information, 2022). The p.Arg167His substitution is located in a functionally significant domain of the myosin head of the protein. The results of pathogenicity prediction for this variant are included in Table S1. This variant should be regarded as having uncertain clinical significance with a level of significance PM2 (moderate piece of evidence for pathogenicity), PP3 (Multiple lines of computational evidence support a deleterious effect on the gene or gene product), and PP4 (phenotype or family history is highly specific for a disease with a single genetic etiology) according to the American College of Medical Genetics and Genomics (ACMG) criteria.

The *MYH14* gene encodes one of the heavy chains of class II non-muscle myosin (NMIIC), a member of the myosin superfamily, and it is a causative gene for autosomal dominant sensorineural hearing loss (AD NSNHL, OMIM #600652, DFNA4). It is widely expressed in the inner ear, including the organ of Corti. *MYH14*-associated hearing loss is rare, the currently available information regarding the variant spectrum and clinical characteristics being limited. There have been reports of 43 *MYH14* variants causing AD NSNHL, most of which were missense mutations (Donaudy et al., 2004; Hiramatsu et al., 2021).

## Conclusions

Our molecular genetic analysis of the causes of NSNHL in the RNO–Alania population revealed pathogenic variants of the *GJB2* gene in 29.7% of the cases, including 26.5% of cases among Ossetians. In Russian patients the most frequent variant was *GJB2*:c.35delG identified in ~83% of the patients. In Ossetian patients, two genetic variants shared more than 91% of the pathogenic alleles of the *GJB2* gene. The most frequent variant in the Ossetian patients was *GJB2*:c.358_360delGAG, which accounted for ~54% (26/48) of the identified pathogenic alleles of the *GJB2* gene. The *GJB2*:c.35delG variant was the second most frequent (~38%, 18/48). The search for large genome rearrangements by MLPA revealed the etiological cause of X-linked hearing loss (type DFNX2), a hemizygous deletion in the *POU3F4* gene locus in two unrelated Ossetian patients. In one of these Ossetian patients, biallelic pathogenic variants in the *MYO15A* gene were associated with DFNB3, and in one Russian family a variant in the *MYH14* gene was associated with autosomal dominant hearing loss of type DFNA4. Thus, the informativity of our molecular genetic diagnostics in the total cohort of NSNHL patients from RNO–Alania was ~37%, or ~32% among Ossetians, which is typical for other populations.

## Supplemental Information

10.7717/peerj.14514/supp-1 Supplemental Information 1 Pathogenicity predictions for the novel single nucleotive variants identified in the study. Each cell indicates the values of the pathogenicity predictions of the novel single nucleotive variants in an annotated vcf format.

10.7717/peerj.14514/supp-2 Supplemental Information 2 Anonymized raw data.

## Additional Information and Declarations

### DNA Deposition

The following information was supplied regarding the deposition of DNA sequences: The mutations revealed in the study are available at ClinVar: VCV000017004, VCV000017006, VCV000017029, VCV000188821, VCV000044766, VCV000044737. The novel variants are available at ClinVar: SUB11631471, SUB11760721.
https://www.ncbi.nlm.nih.gov/clinvar/variation/17004/?redir=vcv

https://www.ncbi.nlm.nih.gov/clinvar/variation/17006/?redir=vcv

https://www.ncbi.nlm.nih.gov/clinvar/variation/17029/?redir=vcv

https://www.ncbi.nlm.nih.gov/clinvar/variation/188821/?redir=vcv

https://www.ncbi.nlm.nih.gov/clinvar/variation/44766/?redir=vcv

https://www.ncbi.nlm.nih.gov/clinvar/variation/44737/?redir=vcv

### Data Availability

The following information was supplied regarding data availability: The data is available in the Supplemental Table.
